# Extracellular membrane microvesicles and nanotubes in the brain: understanding their nature, their function in cell-to-cell communication, their role in transcellular spreading of pathological agents and their therapeutic potential

**DOI:** 10.3389/fphys.2013.00163

**Published:** 2013-07-01

**Authors:** Claudia Verderio

**Affiliations:** ^1^Institute of Neuroscience, Consiglio Nazionale delle RicercheMilan, Italy; ^2^IRCCS HumanitasRozzano, Italy

Although initially perceived as cellular artifacts, nanotubes and extracellular membrane vesicles (EMVs), have been recently recognized as novel pathways for cell-to-cell communication. Membrane nanotubes are long and thin protrusions formed from the plasma membrane that can connect different cells over long distances, up to several micrometers, providing membrane continuity. EMVs are small membranous vesicles which bud directly from the plasma membrane (ectosomes), or result from exocytosis of multivesicular bodies (exosomes). EMVs are capable of transferring specific proteins, lipids, (micro)RNAs and DNAs between cells, thus serving as a unique mechanism for the intercellular trafficking of complex biological messages.

The present Special Issue encompasses thirteen reviews and original articles which outline recent progresses in understanding the biogenesis, biophysical properties and the possible role of nanotubes and EMVs in the healthy and diseased brain. The impressive number of studies accumulated so far indicates that EMVs and nanotubes are becoming of key importance in brain physiology.

The issue opens with a comprehensive review on formation, structure and role of nanotubes in brain cell-to-cell transfer of various signals and materials, including pathogens (Marzo et al., 2012). The attention then turns toward EMVs secreted by neural cells, especially exosomes, their biogenesis and role in inter-neuronal signaling at the synapse, where exosomes may strongly influence plasticity phenomena (Chivet et al., 2012). Three additional review articles broaden the role of neural EMVs to neuron-glia communication in the central and peripheral nervous system. Particular attention is paid to exosomes released by oligodendrocytes and their potential implication in myelin diseases (Frühbeis et al., 2012), to microglia-derived ectosomes and their modulation of excitatory neurotransmission (Turola et al., 2012), and to Schwann cell-derived vesicles and their function in axonal growth and regeneration (Lopez-Verrilli and Court, 2012).

The intriguing capacity of neural EMVs to modulate the immune system activity, either stimulating or repressing, depending on their origin (stem cells, endothelial cells, or tumor cells) is addressed with attention to details by Cossetti et al. (2012). Conversely, the functional activity of EMVs produced by the immune resident brain cells, i.e., microglia, is the focus of a contribution from our own laboratory (Turola et al., 2012).

Among the most relevant effects of EMVs in the diseased brain, D'Asti et al. (2012) highlight the oncogenic properties of tumor-derived EMVs, also called oncosomes, by comprehensively reviewing transformation-related molecules found in their cargo and describing how these effector molecules impact the tumor microenvironment of the central nervous system.

Intriguingly, Bellingham et al. (2012) put forward the hypothesis that neural EMVs may represent a molecular mechanism for the spreading of key proteins involved in neurodegenerative diseases such as, Creutzfeldt-Jakob, Parkinson's and Alzheimer's diseases and amyotrophic lateral sclerosis. The concept that EMVs can deliver and propagate pathogens and misfolded proteins is broaden by Vingtdeux et al. (2012), who specifically discuss the potential contribution of exosomes to amyloid and tau pathologies.

A common issue to the articles focused on brain pathologies is the potential use of EMVs for diagnostics (Bellingham et al., 2012; Colombo et al., 2012; D'Asti et al., 2012). For instance, in brain tumors it is postulated that EMVs circulating in blood or cerebrospinal fluid may be used to decipher molecular features (mutations) of the underlying malignancy and to monitor responses to therapy (D'Asti et al., 2012). While, in neurological disorders with a vascular or ischemic pathogenic component, detection of endothelium- or platelet-derived EMVs in plasma or serum reflects disease activity, and represents a very useful marker to support therapeutic choices (Colombo et al., 2012).

The therapeutical potential of EMVs is instead dual. Indeed blocking EMV secretory pathways could represent a potential therapeutic in neurodegenerative and inflammatory diseases, and in brain tumors (Bellingham et al., 2012; Colombo et al., 2012; D'Asti et al., 2012). On the other hand, the possibility emerges to take advantage of EMVs spreading capability and specificity for drug delivery and therapeutic purposes. This is clearly outlined by Lai and Breakefield (2012), who report emerging EMV-mediated therapies, such as cancer immunotherapy, RNA-interference (RNAi) and drug therapies that could be applied in the foreseeable future to counter brain diseases.

An impressive progress has been recently made in the knowledge of the cellular and molecular mechanisms of EMVs, but still many questions remain to be answered with respect to different aspects of EMV biology. Most EMV functions arise from *in vitro* data obtained in pathological conditions. To evaluate EMV's physiological role during development and adult functions, the field will greatly benefit from the creation of genetic models in which EMV production can be inducibly regulated (Frühbeis et al., 2012; Turola et al., 2012). Moreover, defining EMVs' cargos and understanding EMVs' half-life and circulation *in vivo* will shed light into the intricate intercellular communication system within the body (Lai and Breakefield, 2012). Improvement of isolation protocols, i.e., higher grade of standardization and quality control, and more sensitive and reliable quantification methodologies need to be established by the research community in order to achieve these goals. In this respect, Momen-Heravi et al. (2012a) critically discuss the latest developments in technology concerning methods for EMV isolation and characterization. In addition, the Special Issue ends with an original article describing how viscosity of biological fluids influences isolation efficiency of EMVs by ultracentrifugation, which still represents the “gold standard” method for isolating EMVs (Momen-Heravi et al., 2012b).

Hopefully this Special Issue will encourage/foster innovative studies for the years to come and will stimulate future implications of EMVs and nanotubes in brain functions not yet investigated.
